# Draft Genome Sequence of Fusarium oxysporum f. sp. *albedinis* Strain Foa 133, the Causal Agent of Bayoud Disease on Date Palm

**DOI:** 10.1128/MRA.00462-20

**Published:** 2020-07-16

**Authors:** Slimane Khayi, Siham Khoulassa, Fatima Gaboun, Rabha Abdelwahd, Ghizlane Diria, Mustapha Labhilili, Driss Iraqi, Mohammed El Guilli, Mohamed Fokar, Rachid Mentag

**Affiliations:** a Biotechnology Unit, Regional Center of Agricultural Research of Rabat, National Institute of Agricultural Research (INRA), Rabat, Morocco; b Regional Center of Agricultural Research of Errachidia, National Institute of Agricultural Research (INRA), Errachidia, Morocco; c Regional Center of Agricultural Research of Kénitra, National Institute of Agricultural Research (INRA), Kénitra, Morocco; d Center for Biotechnology and Genomics, Texas Tech University, Lubbock, Texas, USA; Vanderbilt University

## Abstract

Fusarium oxysporum f. sp. *albedinis* is the causal agent of vascular wilt of date palm. Here, we report the genome assembly of the Foa 133 strain, which consists of 3,325 contigs with a total length of 56,228,901 bp, a GC content of 47.42%, an *N*_50_ value of 131,587 bp, and 3,684 predicted genes.

## ANNOUNCEMENT

Fusarium oxysporum f. sp. *albedinis* (Ascomycota, Sordariomycetes, Hypocreales) is the causal agent of date palm *Fusarium* wilt, also known as Bayoud disease (1, 2). It is a very serious fungal disease of the date palm (Phoenix dactylifera L.) (3). Bayoud disease represents a major constraint in the primary date palm growing areas in North African countries, such as Morocco, Algeria, and Mauritania (4–6). The most important means of Bayoud disease transmission are spores and mycelium in the soil. In fact, infection occurs mainly through the roots and spreads internally through the vascular system, leading to wilt and ultimately to date palm death (7, 8). F. oxysporum f. sp. *albedinis* represents a serious threat to date palm biodiversity and productivity, representing a particularly difficult challenge for international trade due to associated quarantine measures (9).

We report the genome sequence of a virulent F. oxysporum f. sp. *albedinis* strain, Foa 133, which was originally isolated from an infected date palm (Khalt) in Tissergate, Morocco (10, 11). Prior to DNA extraction, the fungus was cultivated on potato dextrose agar (PDA) medium, and genomic DNA was extracted from freeze-dried mycelium using the cetyltrimethylammonium bromide (CTAB) method (12). A paired-end library using a Nextera DNA Flex library kit was constructed with total genomic DNA (0.5 μg), following the manufacturer’s user guide, and was sequenced (2 × 150 bp) on a NovaSeq 6000 platform (Illumina, San Diego, CA). In total, 76,022,830 raw reads were obtained. Sequence reads with low quality (limit, 0.05) and ambiguous nucleotides (*n *≤ 2) were removed using CLC Genomics Workbench v12. A total of 70,094,515 (∼10.3 Gb) clean reads were *de novo* assembled using the SPAdes genome assembler v3.14.0 with default parameters (13). The genome size was estimated to be 61 Mbp (61,141,930 bp) based on the 21-mer frequency distribution analysis of the sequence reads using Jellyfish v2.1.4 software (2) and the GenomeScope Web-based application (3). After *de novo* assembly, mitochondrial (MT) sequences corresponding to contig number 234 (length, 51,677 bp) were identified by BLAST search of the assembly against the reference MT sequence from F. oxysporum f. sp. *lycopersici* (GenBank accession number CM010346) and then removed. The nuclear genome of F. oxysporum f. sp. *albedinis* strain Foa 133 consists of 3,325 contigs with a sequencing coverage of 180-fold, an *N*_50_ value of 131,587 bp, 47.42% GC content, and a maximum contig size of 1,175,199 bp as assessed using QUAST (14). The assembled genome reached 91.9% of the estimated size, corresponding to 56,228,901 bp. The completeness of the assembly was assessed with Benchmarking Universal Single-Copy Orthologs (BUSCO) v3.2.1 (15) based on lineage-specific profile library sordariomyceta_odb9, consisting of a set of 3,725 common fungi in a profile library. We retrieved a total of 3,684 complete single BUSCO orthologs (98.9%), 17 fragmented BUSCO orthologs (0.5%), and 24 missing BUSCO orthologs (0.6%). The first draft genome sequence of F. oxysporum f. sp. *albedinis* will provide a useful basis for assessing genetic diversity of this pathogen and to uncover the molecular mechanisms underlying pathogenicity in the F. oxysporum f. sp. *albedinis*-date palm interaction. Furthermore, this sequence will allow comparative genomics studies with other F. oxysporum *formae speciales*.

### Data availability.

This whole-genome shotgun project has been deposited at DDBJ/ENA/GenBank under the accession number JAAVJG000000000. The version described in this paper is version JAAVJG000000000.1. The Illumina reads are available in the SRA under accession number SRR11448459.
